# Identification of Potential Treatments for Acute Lymphoblastic Leukemia through Integrated Genomic Network Analysis

**DOI:** 10.3390/ph15121562

**Published:** 2022-12-14

**Authors:** Zulfan Zazuli, Lalu Muhammad Irham, Wirawan Adikusuma, Nur Melani Sari

**Affiliations:** 1 Department of Pharmacology-Clinical Pharmacy, School of Pharmacy, Bandung Institute of Technology, Bandung 40132, Indonesia; 2 Faculty of Pharmacy, Universitas Ahmad Dahlan, Yogyakarta 55166, Indonesia; 3 Department of Pharmacy, Faculty of Health Science, University of Muhammadiyah Mataram, Mataram 83115, Indonesia; 4 Division of Hematology-Oncology, Department of Child Health, Faculty of Medicine, Universitas Padjadjaran/Dr. Hasan Sadikin General Hospital, Bandung 40161, Indonesia

**Keywords:** acute lymphoblastic leukemia, bioinformatics, leukemia, drug repurposing, genetic variants, genomic network analysis

## Abstract

The advancement of high-throughput sequencing and genomic analysis revealed that acute lymphoblastic leukemia (ALL) is a genetically heterogeneous disease. The abundance of such genetic data in ALL can also be utilized to identify potential targets for drug discovery and even drug repurposing. We aimed to determine potential genes for drug development and further guide the identification of candidate drugs repurposed for treating ALL through integrated genomic network analysis. Genetic variants associated with ALL were retrieved from the GWAS Catalog. We further applied a genomic-driven drug repurposing approach based on the six functional annotations to prioritize crucial biological ALL-related genes based on the scoring system. Lastly, we identified the potential drugs in which the mechanisms overlapped with the therapeutic targets and prioritized the candidate drugs using Connectivity Map (CMap) analysis. Forty-two genes were considered biological ALL-risk genes with *ARID5B* topping the list. Based on potentially druggable genes that we identified, palbociclib, sirolimus, and tacrolimus were under clinical trial for ALL. Additionally, chlorprothixene, sirolimus, dihydroergocristine, papaverine, and tamoxifen are the top five drug repositioning candidates for ALL according to the CMap score with dasatinib as a comparator. In conclusion, this study determines the practicability and the potential of integrated genomic network analysis in driving drug discovery in ALL.

## 1. Introduction

Acute lymphoblastic leukemia (ALL) is not a common disease. However, it is the most common type of cancer in childhood [1]. ALL also represents 75–80% of acute leukemias in children but only 20% in adults [2] and is characterized by proliferation of immature lymphoid cells mainly in the bone marrow and peripheral blood [3]. Although disparities in the region, sex, and age were found, the incidence rate and death rate of ALL were relatively stable during 1990–2017 [3]. During that period, the global incidence case of ALL increased by 30.81% while the age-standardized incidence rate remained stable [3]. ALL-related death also increased by approximately 40.15% worldwide [3]. Due to advancements in the understanding of the molecular genetics and pathophysiological aspects of the disease, risk-adapted treatment algorithms, development of targeted agents, and integration of allogeneic hematopoietic stem cell transplantation (HSCT), the cure rates and survival outcomes for childhood ALL patients have improved significantly over the past several decades [4]. However, there is a subset group that has a proportionally inferior outcome such as the older adolescent and young adult (AYAS) patients, patients with induction failure and relapse in pediatric ALL [5].

Years of research revealed that ALL is a genetically heterogeneous disease. The advancement of high-throughput sequencing and genomic analysis, such as genome-wide association studies (GWAS), have led to numerous studies identifying multiple inherited conditions with predispositions to ALL. ALL can be classified according to the presence of different somatic genetic variations that influence the therapeutic response and overall prognosis [6]. Hence, such information is critical for disease evaluation, optimal risk stratification, and treatment planning.

Certain recurrent genetic abnormalities, such as translocation t(9;22)-*BCR-ABL1* fusion (Ph-positive ALL), hypodiploidy (< 44 chromosomes), *BCR-ABL1*-like (Ph-like) ALL, hyperdiploidy (51–67 chromosomes), t(v;11q23.3) (*KMT2A*-rearranged), t(12;21) (*ETV6-RUNX1*), t(1;19) (*TCF3-PBX1*) and t(5;14) (*IL3-IGH*) represent prognostic factors in children and adolescents with ALL [7,8]. Philadelphia-positive ALL (Ph-positive ALL), which accounts for around 3% of childhood and increases up to 30% as the age of the patients increases, shows a poorer prognosis than other ALL types [7,9]. The incorporation of tyrosine kinase inhibitors (TKIs) such as imatinib significantly improved outcome in Ph-positive ALL patients [10]. Due to the developing imatinib resistance, the second generation TKI dasatinib is also included in the treatment of childhood Ph-positive ALL [7]. However, the rapid development of resistance to TKIs remains a problem that drives the development of new therapies for relapsed/TKI-resistant Ph-positive ALL.

The abundance of such genetic data in ALL can also be utilized to identify potential targets for drug discovery and even drug repurposing. Under such circumstances, ALL-associated genomic variants may ultimately be a good starting point for the implementation of drug repurposing through the concept of genomic-driven drug repurposing. The concept of drug repurposing is proposing an alternative path to utilize the old drug for a new indication. This concept is one of the most promising strategies for translational medicine nowadays [11,12]. In the era of genomic medicine, genomic information (i.e., through GWAS) can be utilized to accelerate the discovery of new indications for old drugs [13]. Several approaches can be applied, including the identification of compounds by linking individual loci to genes and pathways that can be pharmacologically modulated, transcriptome-wide association studies, gene-set association, causal inference by Mendelian randomization, and polygenic scoring [11]. Such an approach can also lead to more effective and targeted drug discovery. However, using high-throughput omics data from multiple studies to further guide effective drug development remains a challenge. Through this study, we aimed to determine potential genes for drug development and further guide the identification of candidate drugs repurposed for treating ALL through integrated genomic network analysis—an in silico-based approach.

## 2. Results

### 2.1. Prioritizing Variants from the GWAS Catalog

Using the predetermined criteria, our search within the GWAS Catalog resulted in 128 hits revealing 74 ALL risk-associated SNPs spreading across 57 known genes (Table 1). Details of search results can be found in Table S1. Expansion of these genes using HaploReg v4.1 resulted in 57 ALL-associated genes.

### 2.2. Prioritizing Biological Risk Gene for ALL

**Figure 1:**
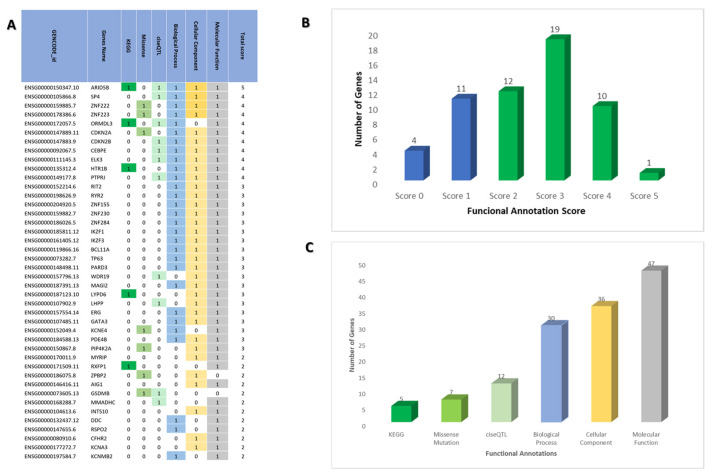
(**A**) List of genes with the score following application of functional annotations criteria. (**B**) The number of genes for each score following application of functional annotations criteria. (**C**) The number of genes overlapped with each functional annotations criterion. Most of the genes overlapped with the molecular function data.

Six biological functional annotations were then applied to 57 ALL-associated genes for further prioritization. Most of the identified genes (*n* = 42; 73.7%) were considered biological ALL-risk genes with scores ≥ 2. We found that 5 out of 57 genes (8.8%) were identified through enrichment analysis in the KEGG database. Missense variations were found in seven genes (12.3%). Cis-eQTL data were found in 12 genes (21.1%) based on whole blood tissue. As many as 52.6%, 63.2%, and 82.5% of genes were supported by biological process, cellular component, and molecular function data, respectively, following integrated analysis using the KEGG and DAVID database. Thus, each gene earned a score based on the number of criteria fulfilled (score ranging from 0 to 6 for each gene). The detailed results can be found in Figure 1. As shown in Figure 1A, the top five biological ALL risk genes include *ARID5B*, *SP4*, *ZNF222*, *ZNF223*, and *ORMDL3*. As shown in Figure 1B, 4 genes scored 0, 11 genes scored 1, and 42 genes with total scores ≥ 2. The 42 genes with a score ≥ 2 were defined as “biological ALL risk genes”. Most of the biological ALL risk genes were scored three (19 genes). *ARID5B* is the most plausible gene due to its high score (score = five).

### 2.3. Drug Target Gene to Be Overlapped with a Drug Database

**Figure 2:**
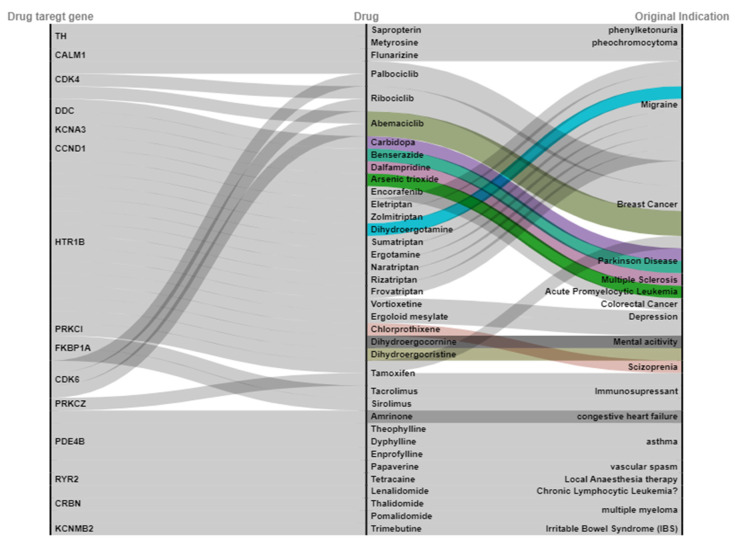
37 drugs overlapped with 15 candidate drug target genes. Four out of these 15 genes (*DDC*, *KCNMB2*, *PDE4B*, and *RYR2*) were included in the list of the previously defined “biological ALL risk genes” (Figure 2). The data visualization in this figure was produced using RAWGraphs visualization (https://app.rawgraphs.io/ accessed on 12 July 2022).

Forty-two biological ALL-risk genes were expanded by using the STRING database. Fifty interactions were selected to perform the calculation and expand the number of genes. After expansion using the STRING database, we generated 92 genes as drug target genes that we regarded as the final list of candidate genes for further analysis. Next, we mapped 92 drug target genes into the DrugBank database with several parameters, such as drugs with pharmacological activity, human efficacy, and annotations of approved, clinical trials or experimental drugs. It is important to note that not all drug target genes can be druggable. Only 15 drug target genes were found to bind to 37 drugs (Figure 2).

### 2.4. Candidate Drug for ALL Undergoing Clinical Trial

**Figure 3:**
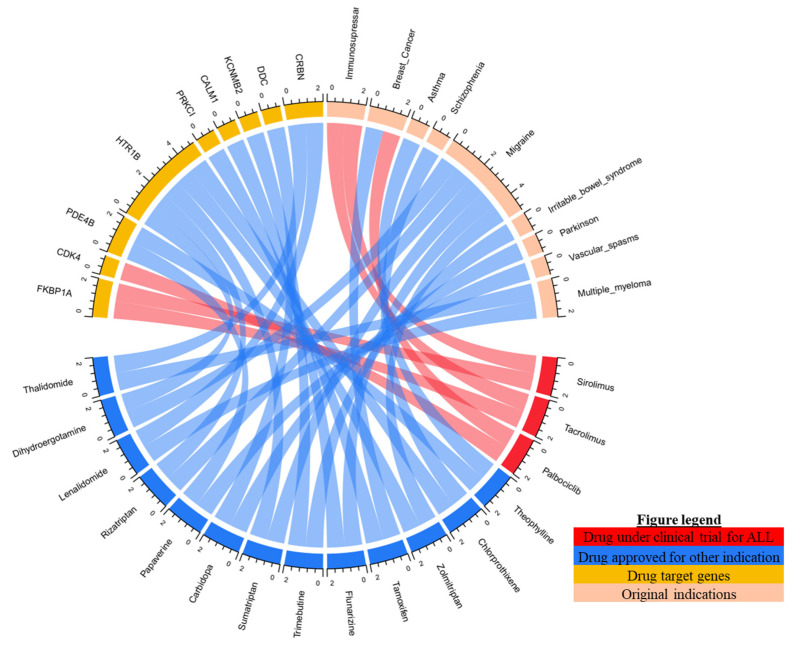
The candidate drugs for acute lymphoblastic leukemia (ALL) are based on CMap analysis.

We further analyzed the drugs listed in Figure 3 to identify the most potential drugs for ALL using CMap analysis. If the two selected drugs (the candidate drugs and dasatinib as a comparator) have a strong positive correlation, the candidate drugs potentially have similar effects in patients with ALL. Intriguingly, we successfully prioritized the list into 16 drugs with 9 candidate drug target genes (Figure 3). We further reviewed the list of drugs using ClinicalTrials.gov (https://clinicaltrials.gov accessed on 13 July 2022) to identify if any clinical research is conducted on the drugs, especially for ALL. Interestingly, we found that three drugs, namely palbociclib, tacrolimus and sirolimus are under clinical trial for ALL. Palbociclib is known as an emerging option for patients with HR+/HER2− advanced or metastatic breast cancer, while tacrolimus and sirolimus are already extensively studied in ALL patients receiving stem cell transplantation.

### 2.5. Candidate Drug for ALL according to CMap Analysis

We further ranked the CMap score of the listed drugs. Chlorprothixene, sirolimus, dihydroergocristine, papaverine, and tamoxifen are the top five drug repositioning candidates when using dasatinib as the comparator with respective CMap scores of 88.76, 87.80, 84.11, 83.98, and 80.92 (Table 2). We found that those drugs also target potential genes that played a role in the development of ALL, such as *HTR1B*, *FKBP1A*, *HTR1B*, *PDE4B*, *PRKC*, and *PCRKCI*. The results suggested an opportunity to repurpose a nonantineoplastic drug as a component for ALL treatments with new target genes.

## 3. Discussion

In this study, we reported potential genes as new drug targets and proposed several drugs potentially to be repositioned for ALL through integrated genomic network analysis. We successfully mined 57 genes that were associated with the increased risk of childhood ALL through the utilization of the GWAS Catalog and expansion of these genes using HaploReg v4.1. To further prioritize the genes which might become potential new drug targets, we scored the genes by applying six functional annotations. Forty-two genes were considered biological ALL risk genes. In line with several metanalyses, we found that *ARID5B* was the most plausible gene [14,15,16].

Noteworthy, *ARID5B*, a member of the AT-rich interactive domain (ARID) protein family, is associated with the incidence and prognosis of ALL, as shown in previous studies [17]. *ARID5B* act as epigenetic regulators by binding with specific or unspecific AT-rich sequences of genomic DNA, and interacting with their partners to modulate chromatin structures. ARID proteins also play an important part in the regulation of development and/or tissue-specific gene expression, especially B-lymphocyte progenitors whose inappropriate expression may enhance tumorigenesis [18,19,20]. Latest study also reported that expression of *ARID5B* varied significantly across ALL subtypes [21]. Variations in this gene also contributed to ethnical disparities in ALL with Hispanic and local native Americans were highly associated [22,23]. A study also showed that compared to healthy bone marrow controls, *ARID5B* is considerably down-regulated in ALL. Low expression of *ARID5B* or *ARID5B* and PHD finger protein 2 (*PHF2*) is correlated with the markers of cell proliferation and poor prognosis in ALL patients. Interestingly, Ikaros, the product of *IKZF* which is essential transcription factor for lymphocyte development and a key suppressor in leukemogenesis, directly regulates *ARID5B* expression in ALL [24]. A study also showed that forced expression of *ARID5B* in immature thymocytes causes thymus retention, differentiation arrest, radioresistance, and tumor development in zebrafish. This is because *ARID5B* is necessary for the survival and expansion of T-ALL cells [25]. Besides its association with the pathogenesis of the disease, *ARID5B* also played a role in ALL prognosis. *ARID5B* knockdown on cell models led to resistance specific to antimetabolites such as 6-mercaptopurine and methotrexate in part through p21-mediated cell-cycle arrest [21]. Additionally, genetic variations in *ARID5B* were associated with serum methotrexate and its metabolite (7-OH-MTX) [26]. It is hypothesized that inherited genetic variations of *ARID5B* SNPs lead to the down-regulation of *ARID5B* expression which further contributes to reduced *ARID5B* expression, blockade of normal lymphocyte development, and finally triggering leukemic clonal expansion [17]. Further studies could be directed to reveal the therapeutic significance of *ARID5B* for the development of ALL treatments.

Following the integration of the STRING dan DrugBank database, we identified 37 drugs that overlapped with 15 candidate drug target genes. Three drugs were already in clinical trials for ALL based on the clinicaltrials.gov database: palbociclib, sirolimus, and tacrolimus. Palbociclib, originally indicated for breast cancer, underwent several phase-I trials on relapse and refractory ALL (NCT03472573, NCT04996160, and NCT03132454) due to its activity on *CDK6*. Since expression of cell cycle regulatory kinase *CDK6* is required for the proliferation and survival of Ph-positive ALL cells, palbociclib could potentially stop the proliferation and accelerate the apoptosis of ALL cells [27]. Sirolimus and tacrolimus which target *FKBP1A* are also studied especially for ALL patients treated with human stem cell transplant (HSCT) [28,29].

Based on CMap database analysis, we listed the top-five drugs potentially to be repurposed for ALL: chlorprothixene, sirolimus, dihydroergocristine, papaverine, and tamoxifen. Surprisingly, only one drug with cancer as the original indication appears on the list. Previous research has reported the potential therapeutic effect of tamoxifen in combination with other therapeutics to enhance the antineoplastic effect of the main drugs. The earliest in vitro study reported that tamoxifen showed cytotoxicity to ALL [30]. Tamoxifen also effectively enhanced the growth-inhibiting actions of various differentiation-inducing agents such as all-trans retinoic acid (ATRA) in acute promyelocytic leukemia cells [31] while the latest research reported that tamoxifen acting on cyclophilin D (CypD) sensitizes T-ALL to mitocans by altering the mitochondrial Ca^2+^ homeostasis [32]. As triphenylethylene antiestrogen, adjuvant tamoxifen with ceramide-centric therapies could increase the therapeutic potential in acute myelogenous leukemia [33]. Additionally, tamoxifen-sensitized Jurkat cells to dexamethasone treatment, which may be related to its capacity to cause autophagy indicating its potential benefit in T-ALL patients [34]. Finally, a phase-I trial of high-dose tamoxifen in combination with daunorubicin in patients with relapsed or refractory acute leukemia suggests that concentrations of tamoxifen high enough to reverse the multidrug-resistant phenotype and that such combination has acceptable toxicity [35]. Our study suggests that tamoxifen targets *PRKCI* that encodes a member of the protein kinase C (PKC) family of serine/threonine protein kinases and is found to be necessary for BCR-ABL-mediated resistance to drug-induced apoptosis and therefore protects leukemia cells against drug-induced apoptosis [36].

Our study also proposed the potential therapeutic benefit of chlorprothixene, a dopamine receptor antagonist, and dihydroergocristine (DHECS), an ergot alkaloid for ALL targeting *HTR1B*. An in silico analysis followed by an in vitro study suggested that chlorprothixene potentially inhibits the growth of acute myeloid leukemia (AML) cells from different subtypes through RNA-seq analysis [37]. Chlorprothixene induced cell cycle, apoptosis, and autophagy in AML cells and inhibited tumor growth, and induced in situ leukemic cell apoptosis in the murine xenograft model [37]. Furthermore, chlorprothixene reduced the level of oncofusion proteins PML-RARα and AML1-ETO led to the elevation of expression of apoptosis-related genes [37]. An in vitro study also reported that DHECS was effective in inducing cell cycle arrest and apoptosis in chemoresistant human prostate cancer cells [38]. Looking at the target gene, the presence of serotonin receptor 1B (*HTR1B*) is needed for AML to progress [39]. Treatment with HTR1B antagonists reduced chronic myeloid clonal neoplasms cell viability and showed a synergistic cytotoxic effect with currently approved hypomethylating agents in AML cells [40]. Another study showed that papaverine which in our analysis targeted *PDE4B*, induces ROS generation, promotes apoptosis, and inhibits Bcr-Abl downstream signaling, thus acting synergistically with the drug imatinib [41]. Phosphodiesterase 4B (*PDE4B*), the main hydrolyzer of cyclic AMP (cAMP) in B cells, was shown to be involved in cell survival and drug resistance in diffuse large B cell lymphomas (DLBCL) [42]. The aforementioned in vitro studies highlighted the potential anticancer properties of non-antineoplastic drugs. Therefore, future functional studies to investigate the mechanisms of how the drugs affect ALL and in vivo studies to strengthen the evidence are needed.

Our study possesses strengths as well as limitations. By using genetic and molecular data on the drugs that are already available in the databases, drug repurposing through such integrated genomic network analysis is a comprehensive resource and a time-efficient process for narrowing down the candidate drugs. Unfortunately, not all the candidates of the drug target genes were druggable. We also noted that the CMap database has not yet included specific transcriptome data from ALL cell lines. Moreover, a study evaluating the performance of CMap reported limited reproducibility in drug repositioning [43]. Thus, standard steps to limit false positives are needed. Finally, the candidate drugs found in this pipeline have not been validated. Nevertheless, further research is required to verify the findings, such as molecular docking, in vivo or in vitro study.

## 4. Materials and Methods

### 4.1. Design

Initially, we conducted the identification of the genomic variant associated with ALL, which was retrieved from the GWAS Catalog. We further applied a genomic-driven drug repurposing approach based on the predetermined criteria to prioritize crucial biological ALL-related genes which were called “biological ALL risk genes”. Such predetermined criteria were needed to filter the candidate genes according to their potential involvement in the trait, as mentioned in the previous publications [44,45]. These genes were suggested to be potential targets for therapeutics in ALL. Lastly, we identified the potential drugs in which the mechanisms overlapped with the therapeutic targets.

### 4.2. Genetic Variants Associated with ALL

**Figure 4:**
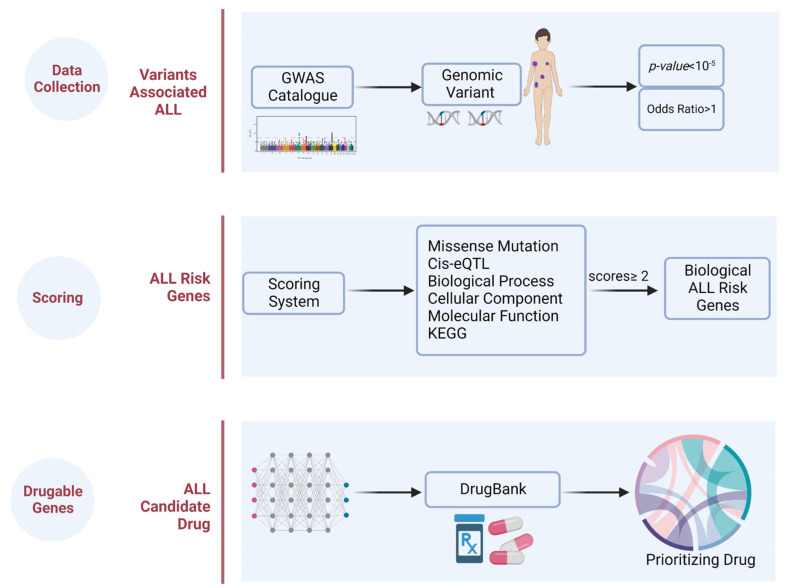
Workflow of the study by leveraging the genomic variants from GWAS and prioritizing it by several functional annotations. Through the genomic variants−driven drug repurposing concept, we finally obtained the drug candidates to be repurposed for ALL according to a drug database.

We identified single nucleotide polymorphisms (SNPs) associated with a higher risk of ALL through multiple GWAS which were curated in one of the largest resources publicly available online (https://www.ebi.ac.uk/gwas/home; accessed on 8 April 2022) [46]. Keywords “acute lymphoblastic leukemia”, “B-cell acute lymphoblastic leukemia”, “childhood acute lymphoblastic leukemia”, and “B-cell acute lymphoblastic leukemia with t(1;19)(q23;p13.3); E2A-PBX1 (TCF3-PBX1) were used to define the traits. The ALL-associated SNPs with *p*-value ≤ 10^−5^ and odds ratio (OR) > 1 were selected. We ascertained that the variants we found are unique SNPs through the removal of duplicate variants. To identify other potential pathways, we expanded our variant search through HaploReg v4.1 (Broad Institute, Cambridge, MA, USA) [47] based on the criterion of linkage disequilibrium (LD) > 0.8. Variants with high LD were then integrated into the 1000 Genome database [48]. It is important to note that the more variants we identified, the more the biological risk gene for ALL we obtained. Again, we removed all SNPs duplicates following the search expansion. Steps on utilizing ALL risk genetic variants to identify biological ALL risk genes and ALL drug target genes were depicted in Figure 4.

### 4.3. ALL Risk Genes

Following search expansion through HaploReg v4.1, SNPs encoded genes were further analyzed to identify the biological risk genes of ALL. We filtered the biological risk genes with strict annotations to determine genes with higher possibility and stronger evidence. In total, six criteria were used in this study to prioritize the biological ALL-risk genes. The genes that fulfilled each criterion will be given one point (maximum six points per gene). The higher the score of a gene, the greater potential of that biological risk gene. We applied the following criteria to filter the biological ALL risk genes: (1) missense mutation, (2) local expression quantitative trait loci (cis-eQTL), (3) biological process, (4) cellular component, (5) molecular function, and (6) the availability of the variants in the Kyoto Encyclopedia of Genes and Genomes (KEGG) [49]. All those criteria were performed by R programming language. Missense mutation encoded the genes were prioritized with the knowledge that functional rules of variants affect protein expression. The missense mutation was integrated using HaploReg v4.1, while the cis-eQTL was integrated using the Genotype-Tissue Expression (GTEx) database [50]. GTEx database is used to study human gene expression and regulation and its relationship to genetic variation. Cis-eQTL represents genomic loci near the gene of origin (a gene that generates the transcript or protein) that contribute to variation in transcript expression levels based on gene expression in whole blood tissue. Gene ontologies include biological processes, cellular components, and molecular functions as criteria 3 to 5 consecutive. To construct gene ontologies, the Database for Annotation, Visualization, and Integrated Discovery (DAVID) online tool version 6.8 was used (https://david-d.ncifcrf.gov/tools.jsp accessed on 10 May 2022). Constructing these gene ontologies aimed to understand the relationship between diseases and biological protein networks. KEGG was integrated with the DAVID database [51] to conduct molecular enrichment analysis, including biological process and molecular function analysis.

### 4.4. Prioritizing the Biological ALL-Risk Genes

Biological ALL-risk genes were prioritized using the scoring system from the six criteria. Genes scored more than equal to 2 were considered biological ALL-risk genes. The more biological ALL-risk genes, the more candidate drug target genes we identified. We proposed the biological ALL-risk genes as candidate drug target genes. Unfortunately, a very limited number of drug target genes are druggable. Therefore, we further expand the biological ALL-risk genes to find more candidate drug target genes using the STRING database [52]. STRING is a database of known and predicted protein–protein interactions, including direct (physical) and indirect (functional) associations. Lastly, we queried the candidate of drug target genes for ALL using the expanded biological ALL-risk genes list.

### 4.5. Drug Identification

To identify the candidate drugs to be repurposed for ALL, we utilized the DrugBank database (www.drugbank.ca (accessed on 12 May 2022). The drug target genes which overlap with the drugs from the DrugBank database will be further proposed for drug repurposing. DrugBank is one of the drug databases and freely accessible databases containing comprehensive molecular information about drugs, their mechanisms, their interactions, and their targets [53].

### 4.6. Connectivity Map (CMap) Analyses

CMap database (https://clue.io/ accessed on 14 May 2022) was utilized to rank the drugs according to a connectivity score ranging from −100 to 100. CMap is a comprehensive catalog of transcriptome data from cultured cells exposed with various chemicals, including drugs. Following a search of intended chemicals, CMap provides a list of small molecules scored to predict their probability to mimic or reverse gene expression profiles of the physiological condition (e.g., diseases). In this study, candidate drugs were compared with dasatinib since tyrosine kinase inhibitor (TKI) is standard therapy for Ph+ ALL patient group (pediatric, adolescent and young adult, and in adults) [2,8]. If the two selected drugs (the candidate drugs and dasatinib as a comparator) have a strong positive correlation (connectivity score > +80), the candidate drugs potentially have similar effects in patients with ALL.

### 4.7. Statistical and Integrated Genomic Analysis

All in-house statistical and genomic database scripts for drug repurposing analysis were written in R programming language (https://www.r-project.org/ accessed on 12 July 2022) using the R Studio 4.0.3 program (RStudio, 250 Northern Ave, Boston, MA, USA). Drug visualization, which integrates the drug target genes and candidate of a new drug to be repurposed for ALL was generated using the RAWGraphs visualization program [54]. While the candidate for drug repurposing based on CMap Analysis was visualized by a chord diagram built using R with the circlize package [55].

## 5. Conclusions

Through integrated genomic network analysis, 42 genes were considered biological ALL-risk genes with *ARID5B* topping the list. Based on potentially druggable genes, palbociclib, sirolimus, and tacrolimus were under clinical trial for ALL. Additionally, chlorprothixene, sirolimus, dihydroergocristine, papaverine, and tamoxifen are the top five drug repositioning candidates. In conclusion, this study determines the practicability and the potential of integrated genomic network analysis in driving drug discovery.

## Figures and Tables

**Table 1:** ALL risk genes that were identified from the GWAS Catalog.

Gene	No. of SNPs	No. of Hits in GWAS Catalog
*IKZF1*	3	13
*ARID5B*	4	12
*GATA3*	1	11
*SLC7A8*, *CEBPE*	2	7
*CDKN2A*	3	4
*LHPP*	3	4
*PIP4K2A*	3	4
*CCDC26*	3	3
*ELK3*	1	3
*GPATCH2L*	1	3
*OR5AL1, OR5AL2P*	1	3
*PDE4B*	3	3
*RNU6-366P, CPSF2*	1	3
*TP63*	1	3
*CSGALNACT1, INTS10*	1	2
*DDC, FIGNL1*	1	2
*ERG*	1	2
*AGBL1*	1	2
*PTPRJ*	1	2
*RN7SL361P*, *BCL11A*	1	2
*RNU6-1091P, IKZF1*	2	2
*RPL6P5*	1	2
Other genes with 1 hit	33	33
Not known genes	2	3
**TOTAL**	74	128

**Table 2:** The top five drug repositioning candidates for acute lymphoblastic leukemia (ALL) were prioritized based on the CMap comparison to dasatinib.

Drugs	Original Indications	Mode of Actions	Drug Target Genes	CMap (Score)
Chlorprothixene	Schizophrenia	inhibitor	*HTR1B*	88.76
Sirolimus	Immunosuppressant	inhibitor	*FKBP1A*	87.80
Dihydroergocristine	Cerebrovascular Diseases	antagonist	*HTR1B*	84.11
Papaverine	Vascular spasm	Inhibitor	*PDE4B*	83.98
Tamoxifen	Breast cancer	inhibitor	*PRKC*, *PRKCI*	80.92

## Data Availability

The data that support the findings of this study are available from the corresponding author, Z.Z., upon reasonable request.

## Supplementary Materials

The following supporting information can be downloaded at: https://www.mdpi.com/article/10.3390/ph15121562/s1, Table S1: ALL risk-associated SNPs generated from GWAS Catalog.

## References

[1] Kaatsch P. (2010). Epidemiology of childhood cancer. Cancer Treat. Rev..

[2] Brown P.A., Shah B., Advani A., Aoun P., Boyer M.W., Burke P.W., DeAngelo D.J., Dinner S., Fathi A.T., Gauthier J. (2021). Acute Lymphoblastic Leukemia, Version 2.2021, NCCN Clinical Practice Guidelines in Oncology. J. Natl. Compr. Cancer Netw..

[3] Yi M., Zhou L., Li A., Luo S., Wu K. (2020). Global burden and trend of acute lymphoblastic leukemia from 1990 to 2017. Aging.

[4] Samra B., Jabbour E., Ravandi F., Kantarjian H., Short N.J. (2020). Evolving therapy of adult acute lymphoblastic leukemia: State-of-the-art treatment and future directions. J. Hematol. Oncol..

[5] Jabbour E., Dull J., Yilmaz M., Khoury J.D., Ravandi F., Jain N., Einsele H., Garcia-Manero G., Konopleva M., Short N.J. (2018). Outcome of patients with relapsed/refractory acute lymphoblastic leukemia after blinatumomab failure: No change in the level of CD19 expression. Am. J. Hematol..

[6] Bloom M., Maciaszek J.L., Clark M.E., Pui C.H., Nichols K.E. (2020). Recent advances in genetic predisposition to pediatric acute lymphoblastic leukemia. Expert Rev. Hematol..

[7] Cerchione C., Locatelli F., Martinelli G. (2021). Dasatinib in the Management of Pediatric Patients With Philadelphia Chromosome-Positive Acute Lymphoblastic Leukemia. Front. Oncol..

[8] Brown P., Inaba H., Annesley C., Beck J., Colace S., Dallas M., DeSantes K., Kelly K., Kitko C., Lacayo N. (2020). Pediatric Acute Lymphoblastic Leukemia, Version 2.2020, NCCN Clinical Practice Guidelines in Oncology. J. Natl. Compr. Cancer Netw..

[9] Liu-Dumlao T., Kantarjian H., Thomas D.A., O’Brien S., Ravandi F. (2012). Philadelphia-positive acute lymphoblastic leukemia: Current treatment options. Curr. Oncol. Rep..

[10] Schultz K.R., Bowman W.P., Aledo A., Slayton W.B., Sather H., Devidas M., Wang C., Davies S.M., Gaynon P.S., Trigg M. (2009). Improved early event-free survival with imatinib in Philadelphia chromosome-positive acute lymphoblastic leukemia: A children’s oncology group study. J. Clin. Oncol.

[11] Kort E., Jovinge S. (2021). Drug Repurposing: Claiming the Full Benefit from Drug Development. Curr. Cardiol. Rep..

[12] Reay W.R., Cairns M.J. (2021). Advancing the use of genome-wide association studies for drug repurposing. Nat. Rev. Genet..

[13] Sanseau P., Agarwal P., Barnes M.R., Pastinen T., Richards J.B., Cardon L.R., Mooser V. (2012). Use of genome-wide association studies for drug repositioning. Nat. Biotechnol..

[14] Yang J.L., Liu Y.N., Bi Y.Y., Wang H. (2019). ARID5B gene polymorphisms and the risk of childhood acute lymphoblastic leukemia: A meta-analysis. Int. J. Hematol..

[15] Zeng H., Wang X.B., Cui N.H., Nam S., Zeng T., Long X. (2014). Associations between AT-rich interactive domain 5B gene polymorphisms and risk of childhood acute lymphoblastic leukemia: A meta-analysis. Asian Pac. J. Cancer Prev..

[16] Guo L.M., Xi J.S., Ma Y., Shao L., Nie C.L., Wang G.J. (2014). ARID5B gene rs10821936 polymorphism is associated with childhood acute lymphoblastic leukemia: A meta-analysis based on 39,116 subjects. Tumour Biol..

[17] Wang P., Deng Y., Yan X., Zhu J., Yin Y., Shu Y., Bai D., Zhang S., Xu H., Lu X. (2020). The Role of ARID5B in Acute Lymphoblastic Leukemia and Beyond. Front. Genet..

[18] Wilsker D., Patsialou A., Dallas P.B., Moran E. (2002). ARID proteins: A diverse family of DNA binding proteins implicated in the control of cell growth, differentiation, and development. Cell Growth Differ..

[19] Webb C.F., Bryant J., Popowski M., Allred L., Kim D., Harriss J., Schmidt C., Miner C.A., Rose K., Cheng H.L. (2011). The ARID family transcription factor bright is required for both hematopoietic stem cell and B lineage development. Mol. Cell Biol..

[20] Yokota T., Kanakura Y. (2014). Role of tissue-specific AT-rich DNA sequence-binding proteins in lymphocyte differentiation. Int. J. Hematol..

[21] Xu H., Zhao X., Bhojwani D., E S., Goodings C., Zhang H., Seibel N.L., Yang W., Li C., Carroll W.L. (2020). ARID5B Influences Antimetabolite Drug Sensitivity and Prognosis of Acute Lymphoblastic Leukemia. Clin. Cancer Res..

[22] Xu H., Cheng C., Devidas M., Pei D., Fan Y., Yang W., Neale G., Scheet P., Burchard E.G., Torgerson D.G. (2012). ARID5B genetic polymorphisms contribute to racial disparities in the incidence and treatment outcome of childhood acute lymphoblastic leukemia. J. Clin. Oncol..

[23] Reyes-Leon A., Ramirez-Martinez M., Fernandez-Garcia D., Amaro-Munoz D., Velazquez-Aragon J.A., Salas-Labadia C., Zapata-Tarres M., Velasco-Hidalgo L., Lopez-Santiago N., Lopez-Ruiz M.I. (2019). Variants in ARID5B gene are associated with the development of acute lymphoblastic leukemia in Mexican children. Ann. Hematol..

[24] Ge Z., Han Q., Gu Y., Ge Q., Ma J., Sloane J., Gao G., Payne K.J., Szekely L., Song C. (2018). Aberrant ARID5B expression and its association with Ikaros dysfunction in acute lymphoblastic leukemia. Oncogenesis.

[25] Leong W.Z., Tan S.H., Ngoc P.C.T., Amanda S., Yam A.W.Y., Liau W.S., Gong Z., Lawton L.N., Tenen D.G., Sanda T. (2017). ARID5B as a critical downstream target of the TAL1 complex that activates the oncogenic transcriptional program and promotes T-cell leukemogenesis. Genes Dev..

[26] Csordas K., Lautner-Csorba O., Semsei A.F., Harnos A., Hegyi M., Erdelyi D.J., Eipel O.T., Szalai C., Kovacs G.T. (2014). Associations of novel genetic variations in the folate-related and ARID5B genes with the pharmacokinetics and toxicity of high-dose methotrexate in paediatric acute lymphoblastic leukaemia. Br. J. Haematol..

[27] Porazzi P., De Dominici M., Salvino J., Calabretta B. (2021). Targeting the CDK6 Dependence of Ph+ Acute Lymphoblastic Leukemia. Genes.

[28] Martinez-Cibrian N., Zeiser R., Perez-Simon J.A. (2021). Graft-versus-host disease prophylaxis: Pathophysiology-based review on current approaches and future directions. Blood Rev..

[29] Paczesny S., Choi S.W., Ferrara J.L. (2009). Acute graft-versus-host disease: New treatment strategies. Curr. Opin. Hematol..

[30] Blatt J., Rotenstein D., Dienes S. (1984). Cytotoxicity of tamoxifen for acute lymphoblastic leukaemia in vitro. Br. J. Cancer.

[31] Adachi K., Honma Y., Miyake T., Kawakami K., Takahashi T., Suzumiya J. (2016). Tamoxifen enhances the differentiation-inducing and growth-inhibitory effects of all-trans retinoic acid in acute promyelocytic leukemia cells. Int. J. Oncol..

[32] Olivas-Aguirre M., Torres-Lopez L., Gomez-Sandoval Z., Villatoro-Gomez K., Pottosin I., Dobrovinskaya O. (2021). Tamoxifen Sensitizes Acute Lymphoblastic Leukemia Cells to Cannabidiol by Targeting Cyclophilin-D and Altering Mitochondrial Ca^(2+)^ Homeostasis. Int. J. Mol. Sci..

[33] Morad S.A., Tan S.F., Feith D.J., Kester M., Claxton D.F., Loughran T.P., Barth B.M., Fox T.E., Cabot M.C. (2015). Modification of sphingolipid metabolism by tamoxifen and N-desmethyltamoxifen in acute myelogenous leukemia--Impact on enzyme activity and response to cytotoxics. Biochim. Biophys. Acta.

[34] Torres-Lopez L., Maycotte P., Linan-Rico A., Linan-Rico L., Donis-Maturano L., Delgado-Enciso I., Meza-Robles C., Vasquez-Jimenez C., Hernandez-Cruz A., Dobrovinskaya O. (2019). Tamoxifen induces toxicity, causes autophagy, and partially reverses dexamethasone resistance in Jurkat T cells. J. Leukoc. Biol..

[35] Berman E., McBride M., Lin S., Menedez-Botet C., Tong W. (1995). Phase I trial of high-dose tamoxifen as a modulator of drug resistance in combination with daunorubicin in patients with relapsed or refractory acute leukemia. Leukemia.

[36] National Center for Biotechnology Information PRKCI Protein Kinase C Iota [Homo Sapiens (Human)]. https://www.ncbi.nlm.nih.gov/gene/5584.

[37] Du Y., Li K., Wang X., Kaushik A.C., Junaid M., Wei D. (2020). Identification of chlorprothixene as a potential drug that induces apoptosis and autophagic cell death in acute myeloid leukemia cells. FEBS J..

[38] Bai L., Li X., Ma X., Zhao R., Wu D. (2020). In Vitro Effect and Mechanism of Action of Ergot Alkaloid Dihydroergocristine in Chemoresistant Prostate Cancer Cells. Anticancer Res..

[39] Galan-Diez M., Borot F., Ali A.M., Zhao J., Gil-Iturbe E., Shan X., Luo N., Liu Y., Huang X.P., Bisikirska B. (2022). Subversion of Serotonin Receptor Signaling in Osteoblasts by Kynurenine Drives Acute Myeloid Leukemia. Cancer Discov..

[40] Banus-Mulet A., Etxabe A., Cornet-Masana J.M., Torrente M.A., Lara-Castillo M.C., Palomo L., Nomdedeu M., Diaz-Beya M., Sole F., Nomdedeu B. (2018). Serotonin receptor type 1B constitutes a therapeutic target for MDS and CMML. Sci. Rep..

[41] Parcha P.K., Sarvagalla S., Ashok C., Sudharshan S.J., Dyavaiah M., Coumar M.S., Rajasekaran B. (2021). Repositioning antispasmodic drug Papaverine for the treatment of chronic myeloid leukemia. Pharmacol. Rep..

[42] Nam J., Kim D.U., Kim E., Kwak B., Ko M.J., Oh A.Y., Park B.J., Kim Y.W., Kim A., Sun H. (2019). Disruption of the Myc-PDE4B regulatory circuitry impairs B-cell lymphoma survival. Leukemia.

[43] Lim N., Pavlidis P. (2021). Evaluation of connectivity map shows limited reproducibility in drug repositioning. Sci. Rep..

[44] Okada Y., Wu D., Trynka G., Raj T., Terao C., Ikari K., Kochi Y., Ohmura K., Suzuki A., Yoshida S. (2014). Genetics of rheumatoid arthritis contributes to biology and drug discovery. Nature.

[45] Irham L.M., Wong H.S., Chou W.H., Adikusuma W., Mugiyanto E., Huang W.C., Chang W.C. (2020). Integration of genetic variants and gene network for drug repurposing in colorectal cancer. Pharmacol. Res..

[46] Buniello A., MacArthur J.A.L., Cerezo M., Harris L.W., Hayhurst J., Malangone C., McMahon A., Morales J., Mountjoy E., Sollis E. (2019). The NHGRI-EBI GWAS Catalog of published genome-wide association studies, targeted arrays and summary statistics 2019. Nucleic Acids Res..

[47] Ward L.D., Kellis M. (2016). HaploReg v4: Systematic mining of putative causal variants, cell types, regulators and target genes for human complex traits and disease. Nucleic Acids Res..

[48] Genomes Project C., Auton A., Brooks L.D., Durbin R.M., Garrison E.P., Kang H.M., Korbel J.O., Marchini J.L., McCarthy S., McVean G.A. (2015). A global reference for human genetic variation. Nature.

[49] Kanehisa M., Furumichi M., Tanabe M., Sato Y., Morishima K. (2017). KEGG: New perspectives on genomes, pathways, diseases and drugs. Nucleic Acids Res..

[50] Consortium G.T. (2020). The GTEx Consortium atlas of genetic regulatory effects across human tissues. Science.

[51] Dennis G., Sherman B.T., Hosack D.A., Yang J., Gao W., Lane H.C., Lempicki R.A. (2003). DAVID: Database for Annotation, Visualization, and Integrated Discovery. Genome Biol..

[52] Szklarczyk D., Gable A.L., Nastou K.C., Lyon D., Kirsch R., Pyysalo S., Doncheva N.T., Legeay M., Fang T., Bork P. (2021). The STRING database in 2021: Customizable protein-protein networks, and functional characterization of user-uploaded gene/measurement sets. Nucleic Acids Res..

[53] Wishart D.S., Feunang Y.D., Guo A.C., Lo E.J., Marcu A., Grant J.R., Sajed T., Johnson D., Li C., Sayeeda Z. (2018). DrugBank 5.0: A major update to the DrugBank database for 2018. Nucleic Acids Res..

[54] Mauri M., Elli T., Caviglia G., Uboldi G., Azzi M. RAWGraphs: A Visualisation Platform to Create Open Outputs. Proceedings of the 12th Biannual Conference on Italian SIGCHI Chapter.

[55] Gu Z., Gu L., Eils R., Schlesner M., Brors B. (2014). circlize Implements and enhances circular visualization in R. Bioinformatics.

