# Supplementary material for: Identification of Potential Treatments for Acute Lymphoblastic Leukemia through Integrated Genomic Network Analysis
Source: Pharmaceuticals (Basel). 2022 Dec 14;15(12):1562. doi: 10.3390/ph15121562 (PMC9786277; doi:10.3390/ph15121562)
Supplement: Supplementary file 1 [file pharmaceuticals-15-01562-s001.zip › pharmaceuticals-1992886-supplementary.pdf]

pharmaceuticals

Supplementary Materials

Identification of Potential Treatments for Acute Lymphoblastic Leukemia through Integrated Genomic Network Analysis

Zulfan Zazuli [1,*], Lalu Muhammad Irham [2], Wirawan Adikusuma [3] and Nur Melani Sari [4]

[1] Department of Pharmacology-Clinical Pharmacy, School of Pharmacy, Bandung Institute of Technology, Bandung 40132, Indonesia
[2] Faculty of Pharmacy, Universitas Ahmad Dahlan, Yogyakarta 55166, Indonesia
[3] Department of Pharmacy, Faculty of Health Science, University of Muhammadiyah Mataram, Mataram 83115, Indonesia
[4] Division of Hematology-Oncology, Department of Child Health, Faculty of Medicine, Universitas Padjadjaran/Dr. Hasan Sadikin General Hospital, Bandung 40161, Indonesia
* Correspondence: zulfan@itb.ac.id; Tel.: +62-222504852

Citation: Zazuli, Z.; Irham, L.M.; Adikusuma, W.; Sari, N.M. Identification of Potential Treatments for Acute Lymphoblastic Leukemia through Integrated Genomic Network Analysis. *Pharmaceuticals* **2022**, *15*, 1562. https://doi.org/10.3390/ph15121562

Academic Editors: Stefania Crucitta, Gloria Ravegnini and Rossana Roncato

Received: 18 October 2022
Accepted: 5 December 2022
Published: 14 December 2022

Publisher's Note: MDPI stays neutral with regard to jurisdictional claims in published maps and institutional affiliations.

Copyright: © 2022 by the authors. Licensee MDPI, Basel, Switzerland. This article is an open access article distributed under the terms and conditions of the Creative Commons Attribution (CC BY) license (https://creativecommons.org/licenses/by/4.0/).

Abstract: The advancement of high-throughput sequencing and genomic analysis revealed that acute lymphoblastic leukemia (ALL) is a genetically heterogeneous disease. The abundance of such genetic data in ALL can also be utilized to identify potential targets for drug discovery and even drug repurposing. We aimed to determine potential genes for drug development and further guide the identification of candidate drugs repurposed for treating ALL through integrated genomic network analysis. Genetic variants associated with ALL were retrieved from the GWAS Catalog. We further applied a genomic-driven drug repurposing approach based on the six functional annotations to prioritize crucial biological ALL-related genes based on the scoring system. Lastly, we identified the potential drugs in which the mechanisms overlapped with the therapeutic targets and prioritized the candidate drugs using Connectivity Map (CMap) analysis. Forty-two genes were considered biological ALL-risk genes with *ARID5B* topping the list. Based on potentially druggable genes that we identified, palbociclib, sirolimus, and tacrolimus were under clinical trial for ALL. Additionally, chlorprothixene, sirolimus, dihydroergocristine, papaverine, and tamoxifen are the top five drug repositioning candidates for ALL according to the CMap score with dasatinib as a comparator. In conclusion, this study determines the practicability and the potential of integrated genomic network analysis in driving drug discovery in ALL.

Keywords: acute lymphoblastic leukemia; bioinformatics; leukemia; drug repurposing; genetic variants; genomic network analysis

Table S1. ALL risk-associated SNPs generated from GWAS Catalog

Variant and risk allele	P-value	P-value annotation	RAF	OR	Beta	CI	Mapped gene	Reported trait	Location
rs282708-**A**	5 x 10-6	(ETV6-RUNX1 positive, Northern European)	0.4	'-	'-	'-	'-	Acute lymphoblastic leukemia (childhood)	4:58637561
rs7738636-**A**	6 x 10-6		0.76	1.2658228	'-	[1.15-1.41]	'-	Acute lymphoblastic leukemia (childhood)	6:77080091
rs282708-**A**	8 x 10-6		0.41	1.23	'-	[1.12-1.35]	'-	Acute lymphoblastic leukemia (childhood)	4:58637561
rs11638062-?	3 x 10-7		'-	1.8	'-	[1.44-2.25]	AGBL1	Acute lymphoblastic leukemia	15:86620033
rs11638062-?	5 x 10-6		NR	2.53	'-	[1.70-3.77]	AGBL1	Acute lymphoblastic leukemia (adult)	15:86620033
rs6901152-**T**	5 x 10-6	(ETV6-RUNX1 positive, Northern European)	0.43	'-	'-	'-	AIG1	Acute lymphoblastic leukemia (childhood)	6:143337875
rs10821936-**C**	1 x 10-106		0.33	1.8	'-	[1.71-1.89]	ARID5B	B-cell acute lymphoblastic leukaemia	10:61963818
rs7089424-?	2 x 10-73		NR	1.8867927	'-	[0.50-0.57]	ARID5B	Acute lymphoblastic leukemia in childhood (B cell precursor)	10:61992400
rs7089424-**T**	2 x 10-62		NR	1.64	'-	[NR]	ARID5B	Acute lymphoblastic leukemia (childhood)	10:61992400
rs7090445-?	5 x 10-54		NR	'-	'-	'-	ARID5B	Acute lymphoblastic leukemia (B-cell precursor)	10:61961417

Variant and risk allele	P-value	P-value annotation	RAF	OR	Beta	CI	Mapped gene	Reported trait	Location
rs10821936-**C**	6 x 10-46		0.33	1.86	'-	[1.71-2.03]	ARID5B	Acute lymphoblastic leukemia (childhood)	10:61963818
rs7089424-**C**	7 x 10-19		0.34	1.65	'-	[1.54-1.76]	ARID5B	Acute lymphoblastic leukemia (childhood)	10:61992400
rs10821936-**C**	1 x 10-15		0.34	1.91	'-	[1.60-2.20]	ARID5B	Acute lymphoblastic leukemia (childhood)	10:61963818
rs10821936-**C**	4 x 10-15		0.31	1.46	'-	[1.33 - 1.60]	ARID5B	Acute lymphoblastic leukemia (childhood)	10:61963818
rs7090445-?	3 x 10-14		NR	1.96	'-	[1.60-2.24]	ARID5B	Acute lymphoblastic leukemia (childhood)	10:61961417
rs10821936-**C**	1 x 10-11	(ETV6-RUNX1 positive)	0.31	1.42	'-	[1.29 - 1.58]	ARID5B	Acute lymphoblastic leukemia (childhood)	10:61963818
rs4245595-**C**	2 x 10-9		0.34	1.63	'-	[1.38–1.93]	ARID5B	Acute lymphoblastic leukemia (childhood)	10:61963136
rs10821936-**C**	2 x 10-8		0.73	2.31	'-	[1.70-3.14]	ARID5B	Acute lymphoblastic leukemia (childhood)	10:61963818
rs4617118-**G**	2 x 10-12		NR	1.28	'-	[1.19-1.37]	CCDC26	Acute lymphoblastic leukemia (childhood)	8:129143897
rs75777619-**G**	2 x 10-9		0.12	1.26	'-	[1.17-1.36]	CCDC26	B-cell acute lymphoblastic leukaemia	8:129172930
rs28665337-?	4 x 10-9		0.12	1.34	'-	[1.21-1.47]	CCDC26	Acute lymphoblastic leukemia in childhood (B cell precursor)	8:129181858
rs113650570-**A**	8 x 10-35		0.02	2.32	'-	[2.03-2.65]	CDKN2A	B-cell acute lymphoblastic leukaemia	9:21976403

Variant and risk allele	P-value	P-value annotation	RAF	OR	Beta	CI	Mapped gene	Reported trait	Location
rs3731249-?	1 x 10-27		NR	2.65	'-	[2.22-3.17]	CDKN2A	Acute lymphoblastic leukemia in childhood (B cell precursor)	9:21970917
rs3731217-?	7 x 10-14		NR	1.4285715	'-	[0.64-0.77]	CDKN2A	Acute lymphoblastic leukemia in childhood (B cell precursor)	9:21984662
rs3731217-?	2 x 10-8		NR	'-	'-	'-	CDKN2A	Acute lymphoblastic leukemia (B-cell precursor)	9:21984662
rs2069426-**A**	4 x 10-9		NR	1.35	'-	[NR]	CDKN2B, CDKN2B-AS1	Acute lymphoblastic leukemia (childhood)	9:22006274
rs4982731-**C**	9 x 10-12		0.28	1.36	'-	[1.24-1.48]	CEBPE, LMLN2	Acute lymphoblastic leukemia (childhood)	14:23116124
rs6428370-**G**	7 x 10-6		0.32	1.43	'-	[1.20-1.60]	CFHR4, CFHR1	Acute lymphoblastic leukemia (childhood)	1:196875463
rs920590-**C**	6 x 10-9	(ETV6-RUNX1 positive, Northern European)	0.31	'-	'-	'-	CSGALNACT1, INTS10	Acute lymphoblastic leukemia (childhood)	8:19793650
rs920590-**C**	2 x 10-6	(ETV6-RUNX1 positive)	0.31	1.19	'-	[1.07 - 1.33]	CSGALNACT1, INTS10	Acute lymphoblastic leukemia (childhood)	8:19793650
rs2167364-**G**	2 x 10-8		0.31	1.32	'-	[1.20 - 1.45]	DDC, FIGNL1	Acute lymphoblastic leukemia (childhood)	7:50498129
rs2167364-**G**	2 x 10-7	(ETV6-RUNX1 positive)	0.31	1.32	'-	[1.19 - 1.46]	DDC, FIGNL1	Acute lymphoblastic leukemia (childhood)	7:50498129

Variant and risk allele	P-value	P-value annotation	RAF	OR	Beta	CI	Mapped gene	Reported trait	Location
rs4762284-**T**	8 x 10-9		0.3	1.19	'-	[1.12–1.26]	ELK3	Acute lymphoblastic leukemia in childhood (B cell precursor)	12:96218984
rs4762284-?	3 x 10-7		NR	1.21	'-	[1.12-1.30]	ELK3	Acute lymphoblastic leukemia in childhood (B cell precursor)	12:96218984
rs4762284-**T**	4 x 10-7		0.32	1.15	'-	[1.12-1.19]	ELK3	B-cell acute lymphoblastic leukaemia	12:96218984
rs6445754-**C**	5 x 10-6	(ETV6-RUNX1 positive, Northern European)	0.22	'-	'-	'-	ERC2	Acute lymphoblastic leukemia (childhood)	3:55773227
rs9976326-**T**	5 x 10-9		0.25	1.33	'-	[1.21-1.46]	ERG	B-cell acute lymphoblastic leukaemia (high-hyperdiploidy)	21:38404563
rs9976326-**T**	1 x 10-8		0.25	1.19	'-	[1.12-1.26]	ERG	B-cell acute lymphoblastic leukaemia	21:38404563
rs3824662-**A**	4 x 10-14		0.19	1.29	'-	[1.21-1.38]	GATA3	B-cell acute lymphoblastic leukaemia	10:8062245
rs3824662-**A**	3 x 10-13		0.2	'-	'-	'-	GATA3	B cell acute lymphoblastic leukaemia (hyperdiploid negative)	10:8062245
rs3824662-**A**	3 x 10-13		0.2	1.9	'-	[1.3-2.7]	GATA3	Acute lymphoblastic leukemia (B-cell precursor)	10:8062245
rs3824662-**A**	1 x 10-12		0.2	'-	'-	'-	GATA3	B cell acute lymphoblastic leukaemia (Philadelphia chromosome negative)	10:8062245

Variant and risk allele	P-value	P-value annotation	RAF	OR	Beta	CI	Mapped gene	Reported trait	Location
rs3824662-**T**	9 x 10-12		0.17	1.31	'-	[1.21-1.41]	GATA3	Acute lymphoblastic leukemia (B-cell precursor)	10:8062245
rs3824662-?	2 x 10-10		NR	1.34	'-	[1.22-1.47]	GATA3	Acute lymphoblastic leukemia in childhood (B cell precursor)	10:8062245
rs3824662-**A**	3 x 10-10		0.20	1.77	'-	[1.48-2.12]	GATA3	Acute lymphoblastic leukemia (adolescents and young adults)	10:8062245
rs3824662-**A**	1 x 10-8		0.2	'-	'-	'-	GATA3	B cell acute lymphoblastic leukaemia (normal cytogenetics)	10:8062245
rs3824662-**A**	2 x 10-7		0.2	'-	'-	'-	GATA3	B cell acute lymphoblastic leukaemia (abnormal cytogenetics)	10:8062245
rs3824662-**A**	2 x 10-7		0.2	2.2	'-	[1.1-4.0]	GATA3	B cell acute lymphoblastic leukaemia in adulthood	10:8062245
rs3824662-**A**	6 x 10-6		0.2	1.9	'-	[1.1-3.3]	GATA3	B cell acute lymphoblastic leukaemia in young adulthood	10:8062245
rs1881797-**C**	7 x 10-6		0.18	1.52	'-	[1.20-1.80]	GCSAML, OR2C3	Acute lymphoblastic leukemia (childhood)	1:247526230
rs7156960-**C**	1 x 10-6	(ETV6-RUNX1 positive)	0.52	1.2048193	'-	[1.09-1.33]	GPATCH2L	Acute lymphoblastic leukemia (childhood)	14:76237008
rs7156960-**C**	3 x 10-6		0.53	1.2195122	'-	[1.12-1.33]	GPATCH2L	Acute lymphoblastic leukemia (childhood)	14:76237008
rs7156960-**G**	4 x 10-6	(ETV6-RUNX1 positive,	0.48	'-	'-	'-	GPATCH2L	Acute lymphoblastic leukemia (childhood)	14:76237008

Variant and risk allele	P-value	P-value annotation	RAF	OR	Beta	CI	Mapped gene	Reported trait	Location
		Northern European)							
rs2290400-**T**	1 x 10-9		NR	1.17	'-	[1.11-1.23]	GSDMB	Acute lymphoblastic leukemia (childhood)	17:39909987
rs4132601-?	6 x 10-55		NR	1.75	'-	[1.63-1.88]	IKZF1	Acute lymphoblastic leukemia in childhood (B cell precursor)	7:50402906
rs11980379-?	3 x 10-33		NR	'-	'-	'-	IKZF1	Acute lymphoblastic leukemia (B-cell precursor)	7:50402283
rs11978267-**A**	2 x 10-29		NR	1.43	'-	[NR]	IKZF1	Acute lymphoblastic leukemia (childhood)	7:50398606
rs4132601-**C**	1 x 10-19		0.28	1.69	'-	[1.58-1.81]	IKZF1	Acute lymphoblastic leukemia (childhood)	7:50402906
rs4132601-**G**	8 x 10-13		0.27	1.43	'-	[1.30 - 1.58]	IKZF1	Acute lymphoblastic leukemia (childhood)	7:50402906
rs11978267-**G**	8 x 10-11		0.27	1.69	'-	[1.40-1.90]	IKZF1	Acute lymphoblastic leukemia (childhood)	7:50398606
rs11978267-**G**	9 x 10-11	(ETV6-RUNX1 positive)	0.27	1.43	'-	[1.28 - 1.59]	IKZF1	Acute lymphoblastic leukemia (childhood)	7:50398606
rs11980379-**C**	1 x 10-9		0.3	2.3	'-	[1.5-3.7]	IKZF1	Acute lymphoblastic leukemia in childhood (B cell precursor)	7:50402283
rs11980379-**C**	4 x 10-9		0.3	'-	'-	'-	IKZF1	B cell acute lymphoblastic leukaemia (Philadelphia chromosome negative)	7:50402283
rs11980379-**C**	4 x 10-8	(males)	NR	1.8	'-	[1.2-2.5]	IKZF1	Acute lymphoblastic leukemia (B-cell precursor)	7:50402283

Variant and risk allele	P-value	P-value annotation	RAF	OR	Beta	CI	Mapped gene	Reported trait	Location
rs11980379-**C**	5 x 10-8		0.3	'-	'-	'-	IKZF1	B cell acute lymphoblastic leukaemia (normal cytogenetics)	7:50402283
rs11980379-**C**	7 x 10-8		0.3	1.5	'-	[1.2-2.0]	IKZF1	Acute lymphoblastic leukemia (B-cell precursor)	7:50402283
rs11980379-**C**	3 x 10-7		0.3	'-	'-	'-	IKZF1	B cell acute lymphoblastic leukaemia (hyperdiploid negative)	7:50402283
rs1110701-**G**	7 x 10-9		0.28	1.69	'-	[1.42–2.02]	IKZF1, RNU6-1091P	Acute lymphoblastic leukemia (childhood)	7:50410929
rs73956024-?	4 x 10-6		NR	3.11	'-	[1.93-5.04]	ISCA1P6, RNA5SP103	Acute lymphoblastic leukemia (adult vs childhood)	2:128513332
rs12621643-**T**	3 x 10-6		0.28	1.48	'-	[1.20-1.70]	KCNE4	Acute lymphoblastic leukemia (childhood)	2:223053265
rs9290663-**T**	6 x 10-6		0.13	1.58	'-	[1.20-1.90]	KCNMB2, KCNMB2-AS1	Acute lymphoblastic leukemia (childhood)	3:178712151
rs10018622-?	4 x 10-6		0.24	1.9607843	'-	[1.45-2.63]	KLHL5, WDR19	Acute lymphoblastic leukemia (childhood)	4:39133944
rs12779301-**C**	6 x 10-13		0.66	1.22	'-	[1.15-1.29]	LHPP	B-cell acute lymphoblastic leukaemia	10:124604086
rs35837782-**G**	1 x 10-11		0.62	1.21	'-	[1.15–1.28]	LHPP	Acute lymphoblastic leukemia in childhood (B cell precursor)	10:124604740
rs35837782-?	9 x 10-11		NR	1.26	'-	[1.18-1.35]	LHPP	Acute lymphoblastic leukemia in childhood (B cell precursor)	10:124604740
rs3740540-**C**	6 x 10-6		NR	1.16	'-	[NR]	LHPP	Acute lymphoblastic leukemia (childhood)	10:124605598

Variant and risk allele	P-value	P-value annotation	RAF	OR	Beta	CI	Mapped gene	Reported trait	Location
rs1496766-**C**	5 x 10-6		0.014	2.84	'-	[1.81-4.44]	MAGI2	Acute lymphoblastic leukemia (childhood)	7:78702149
rs2089222-**A**	8 x 10-8		0.03	2.26	'-	[1.60-3.0]	MAP1LC3B2	Acute lymphoblastic leukemia (childhood)	12:116564853
rs1879352-**C**	9 x 10-6		0.16	1.53	'-	[1.20-1.80]	METTL4	Acute lymphoblastic leukemia (childhood)	18:2498055
rs7578361-**C**	8 x 10-6		0.276	1.4	'-	[1.21-1.63]	MMADHC, LYPD6	Acute lymphoblastic leukemia (childhood)	2:149540704
rs10170236-**C**	4 x 10-6		0.256	1.45	'-	[1.24-1.69]	MMADHC-DT	Acute lymphoblastic leukemia (childhood)	2:149601110
rs17079534-**A**	2 x 10-7		0.005	4.07	'-	[2.40-6.87]	MYRIP	Acute lymphoblastic leukemia (childhood)	3:39805581
rs41322152-**C**	8 x 10-6		0.012	2.52	'-	[1.68-3.79]	NPFFR1	Acute lymphoblastic leukemia (childhood)	10:70281049
rs1945213-**C**	9 x 10-11	(ETV6-RUNX1 positive, Northern European)	0.31	'-	'-	'-	OR5AL1, OR5AL2P	Acute lymphoblastic leukemia (childhood)	11:56408195
rs1945213-**G**	3 x 10-8		0.69	1.2987013	'-	[1.19-1.43]	OR5AL1, OR5AL2P	Acute lymphoblastic leukemia (childhood)	11:56408195
rs1945213-**G**	4 x 10-8	(ETV6-RUNX1 positive)	0.69	1.2820514	'-	[1.14-1.45]	OR5AL1, OR5AL2P	Acute lymphoblastic leukemia (childhood)	11:56408195
rs563507-**A**	9 x 10-6		0.04	2	'-	[1.40-2.70]	PARD3	Acute lymphoblastic leukemia (childhood)	10:34529060

Variant and risk allele	P-value	P-value annotation	RAF	OR	Beta	CI	Mapped gene	Reported trait	Location
rs6683977-**C**	5 x 10-6		0.449	1.41	'-	[1.22-1.64]	PDE4B	Acute lymphoblastic leukemia (childhood)	1:66303417
rs17423910-**G**	6 x 10-6	(ETV6-RUNX1 positive, Northern European)	0.17	'-	'-	'-	PDE4B	Acute lymphoblastic leukemia (childhood)	1:66223426
rs546784-**A**	9 x 10-6		0.457	1.4	'-	[1.20-1.62]	PDE4B	Acute lymphoblastic leukemia (childhood)	1:66296783
rs4748813-**T**	3 x 10-20		NR	1.3513513	'-	[NR]	PIP4K2A	Acute lymphoblastic leukemia (childhood)	10:22557806
rs2296624-**C**	3 x 10-15		0.67	1.25	'-	[1.18-1.32]	PIP4K2A	B-cell acute lymphoblastic leukaemia	10:22568017
rs10828317-?	2 x 10-10		NR	1.2658228	'-	[0.73-0.84]	PIP4K2A	Acute lymphoblastic leukemia in childhood (B cell precursor)	10:22550699
rs10828317-**T**	2 x 10-9		0.68	1.23	'-	[1.15-1.32]	PIP4K2A	Acute lymphoblastic leukemia (B-cell precursor)	10:22550699
rs3942852-**C**	5 x 10-7	(ETV6-RUNX1 positive, Northern European)	0.22	'-	'-	'-	PTPRJ	Acute lymphoblastic leukemia (childhood)	11:48093537
rs3942852-**T**	1 x 10-6	(ETV6-RUNX1 positive)	0.78	1.2987013	'-	[1.12-1.47]	PTPRJ	Acute lymphoblastic leukemia (childhood)	11:48093537

Variant and risk allele	P-value	P-value annotation	RAF	OR	Beta	CI	Mapped gene	Reported trait	Location
rs7142143-**C**	7 x 10-9		0.01	3.61	'-	[2.34-5.57]	PYGL	Acute lymphoblastic leukemia (childhood)	14:50936813
rs9958208-**A**	5 x 10-6		0.099	1.62	'-	[1.32-1.99]	RIT2	Acute lymphoblastic leukemia (childhood)	18:43011119
rs2665658-**A**	2 x 10-8	(EA)	0.35	3.98	'-	[2.46-6.44]	RN7SL361P, BCL11A	TCF3-PBX1 fusion in childhood acute lymphoblastic leukemia	2:60599667
rs2665658-**A**	2 x 10-8	(EA)	0.37	4	'-	[2.47-6.49]	RN7SL361P, BCL11A	Childhood acute lymphoblastic leukemia (TCF3-PBX1 fusion)	2:60599667
rs17133805-**G**	5 x 10-71		0.32	1.65	'-	[1.56-1.74]	RNU6-1091P, IKZF1	B-cell acute lymphoblastic leukaemia	7:50409816
rs6964969-**C**	2 x 10-29		0.28	1.67	'-	[1.53-1.83]	RNU6-1091P, IKZF1	Acute lymphoblastic leukemia (childhood)	7:50405553
rs189434316-**T**	6 x 10-9		0.07	3.7	'-	[2.5-6.2]	RNU6-366P, CPSF2	B cell acute lymphoblastic leukaemia (normal cytogenetics)	14:92231568
rs189434316-**T**	2 x 10-6		NR	'-	'-	'-	RNU6-366P, CPSF2	B cell acute lymphoblastic leukaemia (hyperdiploid negative)	14:92231568
rs189434316-**T**	3 x 10-6		NR	'-	'-	'-	RNU6-366P, CPSF2	B cell acute lymphoblastic leukaemia (Philadelphia chromosome negative)	14:92231568
rs17481869-**A**	2 x 10-9		0.08	1.74	'-	[1.45-2.09]	RPL6P5	B-cell acute lymphoblastic leukaemia (ETV6-RUNX1 positive)	2:145366886
rs17481869-?	5 x 10-6		0.07	1.32	'-	[1.17-1.49]	RPL6P5	Acute lymphoblastic leukemia in childhood (B cell precursor)	2:145366886

Variant and risk allele	P-value	P-value annotation	RAF	OR	Beta	CI	Mapped gene	Reported trait	Location
rs11155133-**G**	3 x 10-7		0.01	3.62	'-	[2.10-6.00]	RPS3AP24	Acute lymphoblastic leukemia (childhood)	6:140848688
rs630662-**A**	2 x 10-6	(ETV6-RUNX1 positive, Northern European)	0.29	'-	'-	'-	RSPO2	Acute lymphoblastic leukemia (childhood)	8:107960070
rs936094-**C**	1 x 10-6	(ETV6-RUNX1 positive, Northern European)	0.17	'-	'-	'-	RXFP1	Acute lymphoblastic leukemia (childhood)	4:158523162
rs7554607-**A**	2 x 10-6		0.56	1.49	'-	[1.20-1.70]	RYR2	Acute lymphoblastic leukemia (childhood)	1:237103303
rs2239630-**A**	2 x 10-21		0.45	1.28	'-	[1.22-1.35]	SLC7A8, CEBPE	B-cell acute lymphoblastic leukaemia	14:23120140
rs2239633-?	1 x 10-16		NR	'-	'-	'-	SLC7A8, CEBPE	Acute lymphoblastic leukemia (B-cell precursor)	14:23119848
rs2239633-?	5 x 10-14		NR	1.369863	'-	[1.28-1.45]	SLC7A8, CEBPE	Acute lymphoblastic leukemia in childhood (B cell precursor)	14:23119848
rs2239633-**T**	7 x 10-13		NR	1.2658228	'-	[NR]	SLC7A8, CEBPE	Acute lymphoblastic leukemia (childhood)	14:23119848
rs2239633-**C**	4 x 10-10		0.5	1.3513513	'-	[1.22-1.47]	SLC7A8, CEBPE	Acute lymphoblastic leukemia (childhood)	14:23119848

Variant and risk allele	P-value	P-value annotation	RAF	OR	Beta	CI	Mapped gene	Reported trait	Location
rs2239633-**C**	2 x 10-8	(ETV6-RUNX1 positive)	0.5	1.3333333	'-	[1.2-1.49]	SLC7A8, CEBPE	Acute lymphoblastic leukemia (childhood)	14:23119848
rs2239633-**G**	3 x 10-7		0.52	1.34	'-	[1.22-1.45]	SLC7A8, CEBPE	Acute lymphoblastic leukemia (childhood)	14:23119848
rs207954-**C**	1 x 10-6	(ETV6-RUNX1 positive)	0.72	1.25	'-	[1.08-1.43]	SLCO3A1	Acute lymphoblastic leukemia (childhood)	15:92114143
rs2390536-**A**	2 x 10-8		NR	1.18	'-	[1.11-1.24]	SP4	Acute lymphoblastic leukemia (childhood)	7:21445779
rs10873876-**T**	4 x 10-6		0.15	1.55	'-	[1.20-1.80]	ST6GALNAC3	Acute lymphoblastic leukemia (childhood)	1:76306643
rs17837497-**A**	2 x 10-6		0.016	2.34	'-	[1.65-3.31]	TBXAS1	Acute lymphoblastic leukemia (childhood)	7:140002794
rs10849033-**G**	9 x 10-6		0.02	2.55	'-	[1.60-3.80]	TIGAR	Acute lymphoblastic leukemia (childhood)	12:4315956
rs17505102-**G**	9 x 10-9	(ETV6-RUNX1 positive)	0.85	1.5873016	'-	[1.33-1.92]	TP63	Acute lymphoblastic leukemia (childhood)	3:189683987
rs17505102-**G**	2 x 10-8		0.85	1.4705882	'-	[1.28-1.67]	TP63	Acute lymphoblastic leukemia (childhood)	3:189683987
rs17505102-**C**	3 x 10-8	(ETV6-RUNX1 positive, Northern European)	0.15	'-	'-	'-	TP63	Acute lymphoblastic leukemia (childhood)	3:189683987

Variant and risk allele	P-value	P-value annotation	RAF	OR	Beta	CI	Mapped gene	Reported trait	Location
rs343604-**T**	1 x 10-6	(ETV6-RUNX1 positive, Northern European)	0.08	'-	'-	'-	Y_RNA, KCNA3	Acute lymphoblastic leukemia (childhood)	1:110716148
rs2191566-**G**	4 x 10-7		0.28	1.52	'-	[1.20-1.70]	ZNF230	Acute lymphoblastic leukemia (childhood)	19:44007237
